# Factors Influencing Treatment Dropout Among Child and Adolescent Psychiatric Patients: A Survival Analysis

**DOI:** 10.1007/s10880-026-10159-8

**Published:** 2026-06-11

**Authors:** Yoshinori Sasaki, Masahide Usami, Yuki Hakosima, Kumi Inazaki, Yuki Mizumoto, Masaya Ito, Katsunaka Mikami, Noa Tsujii, Takayuki Okada, Hidehiko Takahashi

**Affiliations:** 1 Institute of Science Tokyo Graduate School, Tokyo, Japan; 2 Kohnodai Hospital, National Center for Global Health and Medicine, Chiba, Japan; 3 Kohnodai Hospital, National Center for Global Health and Medicine, Chiba, Japan; 4 National Center of Neurology and Psychiatry, Kodaira, Japan; 5 Tokai University School of Medicine, Kanagawa, Japan; 6 Toyama University Hospital, Toyama, Japan; 7 Institute of Science, Tokyo Centre for Brain Integration Research, Institute of Biomedical Engineering, Tokyo, Japan

**Keywords:** Treatment dropouts, Child psychiatry, Survival analysis, Divorce, Drug prescriptions

## Abstract

Child and adolescent mental health disorders are a significant global health concern, with high rates of treatment dropout adversely affecting long-term outcomes. This retrospective cohort study aimed to identify factors influencing treatment dropout in child and adolescent psychiatric patients. 443 outpatients aged < 15 years who visited a specialized child and adolescent psychiatry outpatient clinic at a national medical center in Japan between April 2022 and March 2023 were followed for 1 year. Dropout was defined as failure to attend scheduled appointments without notice or having no record of visits for more than 6 months after the last visit. Of the 443 patients, 74 (16.7%) dropped out within 1 year. Parental divorce or separation increased dropout risk (odds ratio [OR] = 2.28, 95% confidence interval [CI]: 1.05–5.10, p < 0.05), whereas receiving a prescription at the initial visit decreased risk (OR = 0.27, 95% CI: 0.10–0.76, p < 0.05; hazard ratio = 0.28, 95% CI: 0.12–0.65, p < 0.01). The Kaplan–Meier analysis revealed higher 1-year retention rates for patients receiving initial prescriptions (93 vs. 79%), particularly among females (98 vs. 79%). Early pharmacological interventions and identification of high-risk family constellations may enhance treatment retention in child and adolescent psychiatry.

## Introduction

Child and adolescent mental health disorders represent a significant global health concern, with consistently high prevalence rates reported across various studies. Recent epidemiological data indicate that approximately 13.4% of children and adolescents worldwide have mental disorders (Polanczyk et al., 2015). The prevalence increases to nearly 20% when considering a broad range of mental, emotional, developmental, or behavioral disorders (Centers for Disease Control & Prevention, 2025). Longitudinal studies suggest that up to 36.7% of individuals meet the criteria for at least one mental disorder by the age of 18 years (Costello et al., 2003). Moreover, prospective cohort data indicate that the cumulative prevalence of psychiatric disorders reaches over 60% by young adulthood, underscoring the importance of early identification and intervention (Copeland et al., 2011). The most common conditions include anxiety disorders (5.2%), attention-deficit/hyperactivity disorders (3.7%), and oppositional defiant disorders (3.3%) (Barican et al., 2022). Notably, half of all mental illnesses begin by the age of 14 years, highlighting the importance of early identification and intervention (Gupta et al., 2024). These disorders not only affect immediate well-being but also have long-term consequences, significantly contributing to the global burden of the disease in this age group (US Agency for Healthcare Research and Quality, 2022; Piao et al., 2022). The prevalence of mental health disorders in children and adolescents has shown an alarming increase in recent decades, with some studies reporting an increase from 5.4% in 2003 to 8.4% in 2011–2012 for depression and anxiety in the United States (Hossain et al., 2022).

Treatment continuity and adherence are critical factors in the management of child and adolescent psychiatric disorders, as treatment dropout represents a severe form of non-adherence and has been linked to poorer clinical outcomes (Gajria et al., 2014; Timlin et al., 2015; Wong et al., 2009). Approximately 50% of patients fail to adhere to treatment guidelines or discontinue treatment within 2–3 years after initiating pharmacological therapy (Gajria et al., 2014). The consequences of non-adherence can be severe, including an increased risk of psychiatric disorder recurrence and suicidality in adulthood (Wong et al., 2009). Additionally, low adherence contributes to increased morbidity and medical complications, ultimately diminishing overall quality of life (Timlin et al., 2015).

Recent studies have identified multiple factors associated with treatment dropout in child and adolescent psychiatry, which can be broadly categorized into patient-, family-, treatment-, and system-level factors (de Haan et al., 2013; de Soet et al., 2024; Kazdin & Mazurick, 1994). At the patient level, older age and the presence of externalizing disorders or substance use disorders are associated with higher dropout rates, whereas children with mood disorders tend to show better treatment adherence (de Soet et al., 2024; Kazdin & Mazurick, 1994; Wong et al., 2009). At the family level, parental divorce or separation, family conflict, and caregivers’ mental health problems are known to impede treatment continuity, and parental attitudes and support play a key role in maintaining adherence (Alsubaie et al., 2024; Johnson et al., 2008; Kazdin & Mazurick, 1994; Wong et al., 2009). At the treatment level, medication side effects, complex treatment plans, and poor therapeutic alliance between the patient, family, and provider have been linked to dropout (Khan & Aslani, 2020; Timlin et al., 2015). Notably, receiving a prescription at the initial visit has been associated with lower dropout rates (Brikell et al., 2021). At the system level, low socioeconomic status and limited access to care further complicate treatment adherence (Kazdin, 1990; Kazdin & Mazurick, 1994). Despite this growing body of evidence, knowledge gaps remain regarding the relative contribution of these factors across different psychiatric diagnoses and treatment modalities in children and adolescents (de Haan et al., 2013). In particular, studies simultaneously examining patient-, family-, and treatment-level predictors in a diagnosis-transcending, real-world outpatient sample are scarce.

This study aimed to identify and analyze the factors influencing treatment dropout among children and adolescent psychiatric patients. Based on the existing literature and clinical observations, we hypothesized that factors such as age, sex, diagnoses, parental divorce or separation, suicide-related behaviors, school refusal, abuse history, and initial prescription status influence treatment adherence. This study aims to identify and analyze factors associated with treatment dropout among child and adolescent psychiatric outpatients. By clarifying which patient-, family-, and treatment-level factors predict dropout, the findings could inform the development of targeted clinical strategies—such as enhanced support for patients from high-risk family backgrounds and early engagement interventions at the initial visit—that may ultimately contribute to improved treatment retention and long-term outcomes in this vulnerable population.

## Methods

### Study Design

This study was conducted in accordance with the STROBE guidelines. This retrospective cohort study analyzed data from child and adolescent psychiatric outpatients who initially visited the Department of Child and Adolescent Psychiatry at a specialized child and adolescent psychiatry outpatient clinic of a national medical center in Japan between April 1, 2022, and March 31, 2023. Each patient was tracked for 1 year from their initial visit to determine their treatment status (dropout, transfer, completion, continued outpatient care, or hospitalization) over a full year of potential treatment. The primary outcome measure was to identify and analyze factors influencing treatment dropout, defined as cases where patients had scheduled appointments but failed to attend without prior notice or explanation, or had no record of visits for more than 6 months after their last visit within 1 year of the initial visit (case group). Patients who missed a scheduled appointment but subsequently returned to treatment within 6 months were not classified as dropouts. Patients who did not drop out of treatment (control group) included: those who were transferred to another medical facility (e.g., because of relocation or patient/family preference); those who achieved treatment completion within the 1-year follow-up period (i.e., treatment was planned and concluded after achieving therapeutic goals, following consultation between the doctor, patient, and family); those who continued outpatient care, including patients who shifted from pharmacotherapy to psychotherapeutic or psychosocial interventions within the same clinic (such patients were not classified as transfers, as they remained under the care of the same institution); and those who were hospitalized. This research was facilitated by weekly reviews of appointments and actual consultation records.

### Study Population

This study was conducted at a specialized child and adolescent psychiatric outpatient clinic in Japan. In this clinic, all prescriptions and medication refills are issued during follow-up consultations conducted by child and adolescent psychiatrists. This study included child and adolescent psychiatric outpatients aged < 15 years at their initial visits. Patients with moderate to severe intellectual disability, organic brain disease, drug-induced psychiatric disorders, traumatic brain injury, or genetic syndromes were excluded, as they are typically referred to other medical institutions at their first appointment.

### Measures

At the initial visit, psychologists and psychiatrists interviewed the patients and their parents to assess their demographic and clinical characteristics. The sex and age of the patients were recorded at this time. Additionally, the prescription status was recorded to check whether any psychiatric medication was prescribed at the initial visit. Information on parental divorce or separation was collected to understand family dynamics. This variable was assessed at the initial visit and treated as a baseline characteristic; changes in family status during the follow-up period were not systematically recorded. Suicide-related behavior was defined broadly as any past or current self-injurious behavior or overdose recorded as being related to suicidality, including suicide attempts. Because non-suicidal self-injury and suicidal self-injury were not systematically differentiated in the clinical records, both types of behavior were included in this category. School refusal was defined as absence from school for ≥ 30 d during an academic year due to psychological, emotional, physical, or social factors (Ministry of Education, Culture, Sports, Science, and Technology, 2024). The abuse history was evaluated by an attending psychiatrist based on comprehensive interviews and available information. Furthermore, the treating psychiatrists interviewed parents and patients separately to increase the likelihood of identifying possible cases of abuse. Diagnoses were made in accordance with the International Classification of Diseases, Tenth Revision Classification of Mental and Behavioral Disorders (World Health Organization, 1992). In routine clinical practice, case conferences focused on new patients are held regularly, and diagnoses are confirmed through discussions among child and adolescent psychiatrists, clinical psychologists, social workers, and other team members.

### Statistical Analysis

We employed a comprehensive approach to examine the factors associated with treatment dropout. Descriptive statistics were used to summarize the characteristics of the study population. A case–control comparison was conducted between the dropout and non-dropout groups. Pearson’s chi-squared and Fisher’s exact tests were used for categorical variables, whereas the Mann–Whitney U test was applied for continuous variables, accounting for potential non-normal distributions. To further explore these associations, a multivariate logistic regression analysis was conducted, including variables such as sex, age, parental divorce or separation, suicide-related behavior, school refusal, prescription at the initial visit, and abuse history. The core of the analysis was the survival analysis technique. Kaplan–Meier survival curves were generated to visualize cumulative dropout-free survival over time and calculate the proportion of individuals who remained in contact with medical institutions over the course of 1 year. Log-rank tests were used to statistically compare the survival curves between groups. Cox proportional hazard models were used to assess the impact of multiple variables on dropout risk while adjusting for potential confounders. For variables identified as significant in the Cox regression, additional Kaplan–Meier curves stratified using these factors were generated to illustrate their impact on dropout rates over time. All statistical tests were two-tailed, with p < 0.05 considered statistically significant. All analyses were performed using SPSS version 27.0 (IBM, Armonk, NY). This comprehensive approach integrates both case–control and time-to-event analytical frameworks.

## Results

### Participants and Descriptive Data

The study included 443 patients who had their first visit to a specialized child and adolescent psychiatry outpatient clinic of a national medical center in Japan between April 1, 2022 and March 31, 2023. These patients were monitored for 1 year to track their clinical course. During this period, the following outcomes were observed: treatment dropout (n = 74), transfer to another medical facility (n = 34), treatment completion after achieving therapeutic goals (n = 47), continued outpatient care (n = 273), and hospitalization (n = 15).

### Outcome Data

Table 1 compares the clinical characteristics of the case (N = 74) and control groups (N = 369). A significantly higher proportion of patients in the control group received prescriptions at their initial visit compared to that in the case group (23.8 vs. 8.1%, p < 0.01). The proportion of F3 diagnostic classification for mood disorders was significantly higher in the control group (9.5 vs. 2.7%, p < 0.05) than in the case group. No significant differences were observed in other characteristics, including male sex, age, parental divorce or separation, suicide-related behavior, school refusal, or abuse history (Table 1).

**Table 1:** Comparison of the clinical characteristics expressed as percentages of binary and continuous variables for case versus control groups

Characteristics	Case (N = 74) % (n)	Control (N = 369) % (n)	X^2^	df	Effect Size	p value
Male sex	54.1 (40)	47.4 (175)	1.08	1	0.05	0.30
Age (years)	11.00 ± 3.39^a^	10.75 ± 3.08^a^	–	–	0.05	0.29
Any prescription at initial visit	8.1 (6)	23.8 (88)	9.13	1	0.14	** < 0.01**
Divorce or separation of parents	24.4 (11)	18.9 (25)	0.63	1	0.12	0.43
Suicide-related behavior	14.9 (11)	9.8 (36)	1.68	1	0.06	0.20
School refusal	41.9 (31)	39.7 (146)	0.13	1	0.02	0.72
Abuse history	13.5 (10)	9.2 (34)	1.26	1	0.05	0.26
Diagnostic classification						
F0	0 (0)	1 (0.3)	–^b^	–^b^	0.02	0.65
F2	0 (0)	4 (1.1)	–^c^	–^c^	0.04	0.37
F3	7 (9.5)	10 (2.7)	–^d^	–^d^	0.13	** < 0.05**
F4	19 (25.7)	120 (32.5)	1.34	1	0.25	0.25
F5	5 (6.8)	33 (8.9)	0.38	1	0.03	0.54
F6	2 (2.7)	5 (1.4)	–^e^	–^e^	0.04	0.33
F7	6 (8.1)	25 (6.8)	0.17	1	0.02	0.68
F8	24.3 (18)	25.2 (93)	0.25	1	0.01	0.87
F9	23.0 (17)	20.9 (77)	0.16	1	0.02	0.69

^a^mean ± standard deviation

Due to the low expected frequency ^b^0.17, ^c^0.67, ^d^2.84 and ^e^1.17 < 5, Fisher’s exact test was applied, and no Pearson’s chi-squared value was calculated

Effect sizes are reported as Cohen’s d for continuous variables and Cramer’s V for categorical variables

F0 = Organic, including symptomatic, mental disorders

F2 = Schizophrenia, schizotypal and delusional disorders

F3 = Mood disorders

F4 = Neurotic, stress-related and somatoform disorders

F5 = Behavioral syndromes associated with physiological disturbances and physical factors

F7 = Mental retardation

F8 = Disorders of psychological development (e.g., autism spectrum disorder)

F9 = Behavioral and emotional disorders with onset usually occurring in childhood and adolescence (e.g., attention-deficit/hyperactivity disorder)

Statistically significant values were boldface

The results of the multivariate logistic regression analysis are presented in Table 2. Parental divorce or separation was significantly associated with an increased likelihood of dropout (case group), with an odds ratio (OR) of 2.28 (95% confidence interval [CI]: 1.05–5.10, p < 0.05). Conversely, receiving a prescription at the initial visit was associated with a decreased likelihood of dropout (case group), with an OR of 0.27 (95% CI: 0.10–0.76, p < 0.05). Other variables, including male sex, age, suicide-related behavior, school refusal, and history of abuse, were not significant predictors (Table 2).

**Table 2:** Results of the multivariate logistic regression analyses

Characteristics	OR	95% CI	p value
Male sex	1.60	0.85–3.03	0.15
Age	1.03	0.92–1.16	0.58
Any prescription at initial visit	0.27	0.10–0.76	** < 0.05**
Divorce or separation of parents	2.28	1.02–5.10	** < 0.05**
Suicide-related behavior	1.83	0.71–4.70	0.21
School refusal	1.10	0.55–2.20	0.79
Abuse history	1.19	0.42–3.32	0.75

Each parameter was adjusted for male sex, age, divorce or separation of parents, suicide-related behavior, school refusal, any prescription at initial visit, and abuse history

Statistically significant values are shown in bold. OR, odds ratio; CI, confidence interval

Table 3 demonstrates the factors associated with dropout rates based on the Kaplan–Meier survival analysis. Patients whose parents were divorced or separated showed a significantly higher dropout risk compared to those without parental divorce or separation (hazard ratio [HR] = 1.90, 95% CI: 1.13–3.20, p < 0.05). Additionally, patients who received any prescription at the initial visit had a significantly lower dropout risk compared to those who did not receive a prescription (HR = 0.30, 95% CI: 0.13–0.69, p < 0.01). No significant associations were observed with sex, age, suicide-related behavior, school refusal, or abuse history (Table 3).

**Table 3:** Univariate analyses of the characteristics of dropout patients

Characteristics	Mean (SD) or n (%)	1-year connected to medical institutions estimate^a^	Hazard ratio^b^	95% CI^c^	χ^2^	p value
Sex Male Female	215 (48.5) 228 (51.5)	0.80 0.83	1.23	0.78–1.94 (Reference category)	0.79	0.38
Age ≥ 12 years ≤ 11 years	226 (51.0) 217 (49.0)	0.80 0.83	1.25	0.79–1.97 (Reference category)	0.89	0.35
Any prescription at initial visit Yes No	94 (21.2) 349 (78.8)	0.93 0.78	0.30	0.13–0.69 (Reference category)	9.10	** < 0.01**
Divorce or separation of parents Yes No	71 (16.0) 372 (84.0)	0.71 0.84	1.90	1.13–3.20 (Reference category)	6.02	**0.02**
Suicide-related behavior Yes No	47 (11.9) 395 (88.1)	0.73 0.83	1.53	0.81–2.90 (Reference category)	1.72	0.19
School refusal Yes No	177 (40.0) 265 (60.0)	0.81 0.82	1.08	0.68–1.71 (Reference category)	0.10	0.76
Abuse history Yes No	44 (12.9) 398 (88.1)	0.74 0.82	1.51	0.77–2.93 (Reference category)	1.47	0.23

^a^From Kaplan–Meier survival function

^b^Hazard ratio is change relative to the reference category

^c^CI = confidence interval

Statistically significant values are shown in bold

The results of the multivariate Cox regression model are presented in Table 4. Patients who received a prescription at the initial visit had a significantly lower risk of dropout than those who did not receive a prescription. The dropout risk was 0.28 times lower for patients with an initial prescription (HR = 0.28, 95% CI: 0.12–0.65, p < 0.01). No other variables, including sex, age, parental divorce or separation, suicide-related behavior, school refusal, or history of abuse, were significant predictors of dropout risk (Table 4).

**Table 4:** Multivariate model of dropout risk

Characteristics	Hazard ratio	95% CI	Wald	p value
Sex^a^	1.37	0.85–2.23	1.67	0.20
Age^b^	1.34	0.79–2.27	1.18	0.28
Any prescription at initial visit^a^	0.28	0.12–0.65	8.85	** < 0.01**
Divorce or separation of parents^a^	1.73	0.96–3.13	3.35	0.07
Suicide-related behavior^a^	1.59	0.81–3.11	1.83	0.18
School refusal^a^	0.95	0.57–1.59	0.03	0.86
Abuse history^a^	1.24	0.59–2.63	0.32	0.57

^a^Categorical variable

^b^Continuous variable

Statistically significant values are shown in bold. CI, confidence interval

**Fig. 1:**
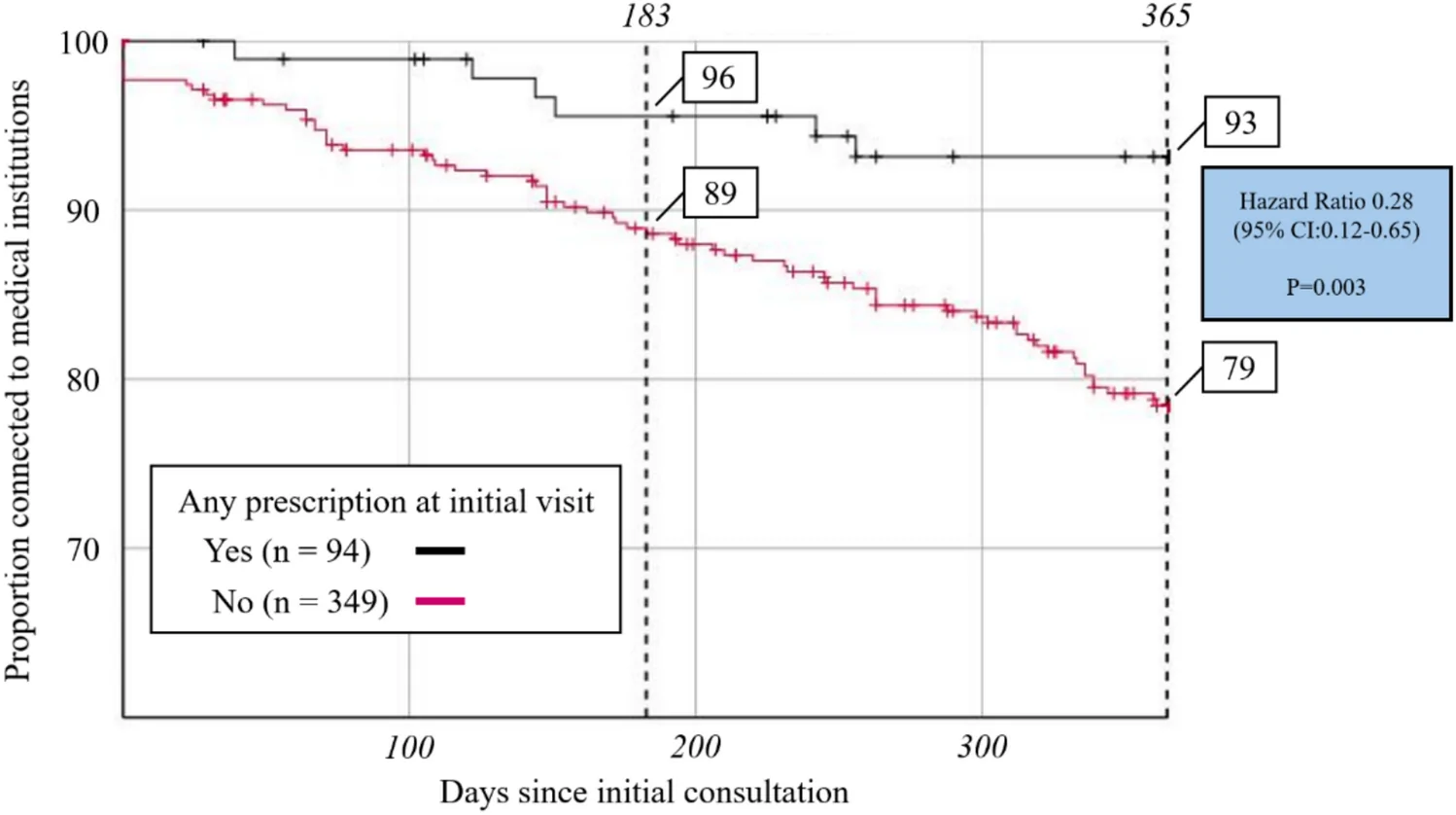
Kaplan–Meier survival curve for the period from treatment to dropout (total). CI, confidence interval

The Kaplan–Meier survival curve for all patients, illustrating the period from initial treatment to dropout, is shown in Fig. 1. A significant difference was observed between patients who received any prescription at the initial visit and those who did not (HR = 0.28, 95% CI: 0.12–0.65, p = 0.003).

**Fig. 2:**
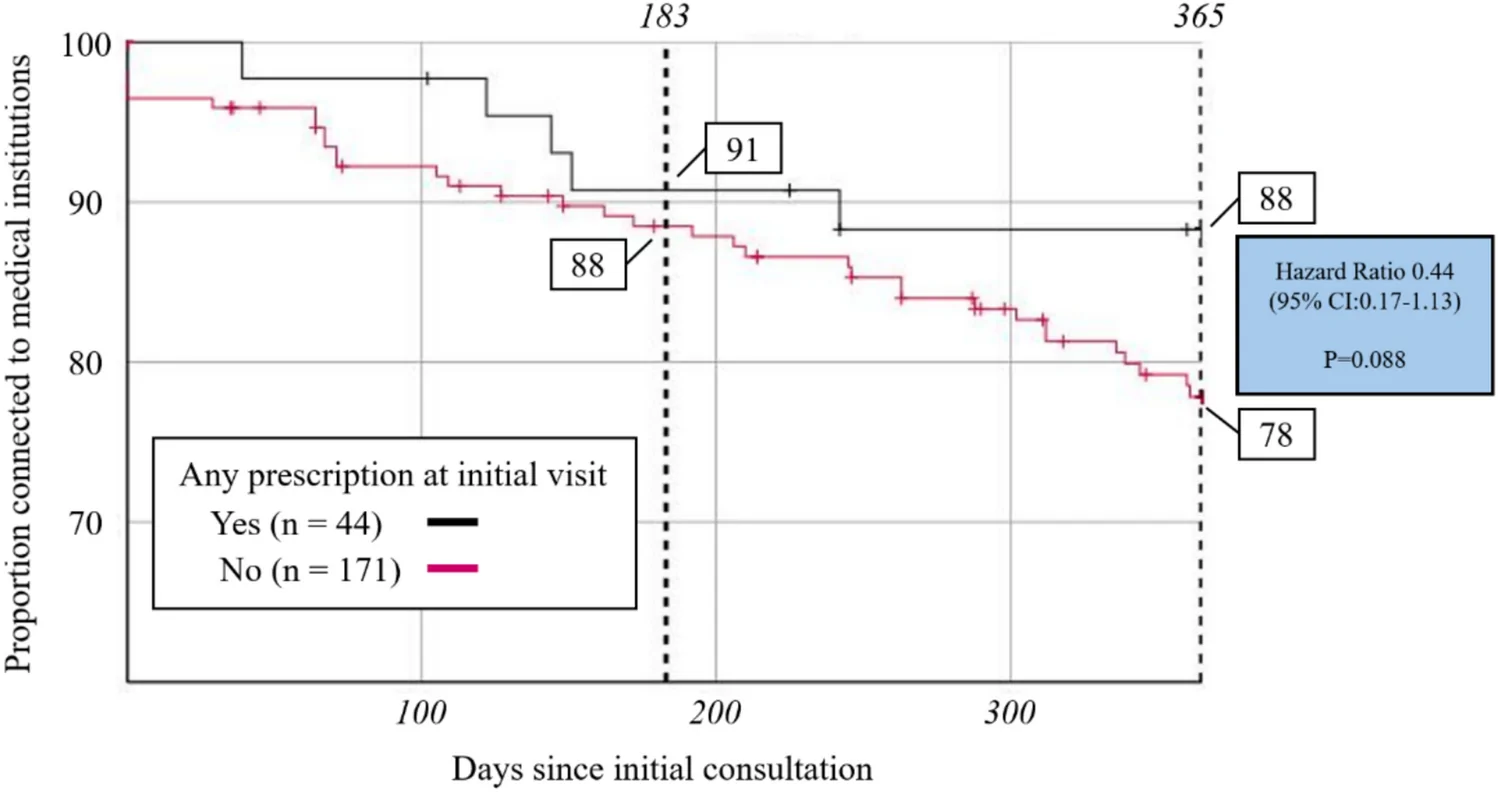
Kaplan–Meier survival curve for the period from treatment to dropout (male). CI, confidence interval

Figure 2 presents the Kaplan–Meier survival curve for male patients from initial treatment to dropout. The graph indicates a difference between those who received any prescription at the initial visit and those who did not, although this difference was not statistically significant (HR = 0.44, 95% CI: 0.17–1.13, p = 0.088).

**Fig. 3:**
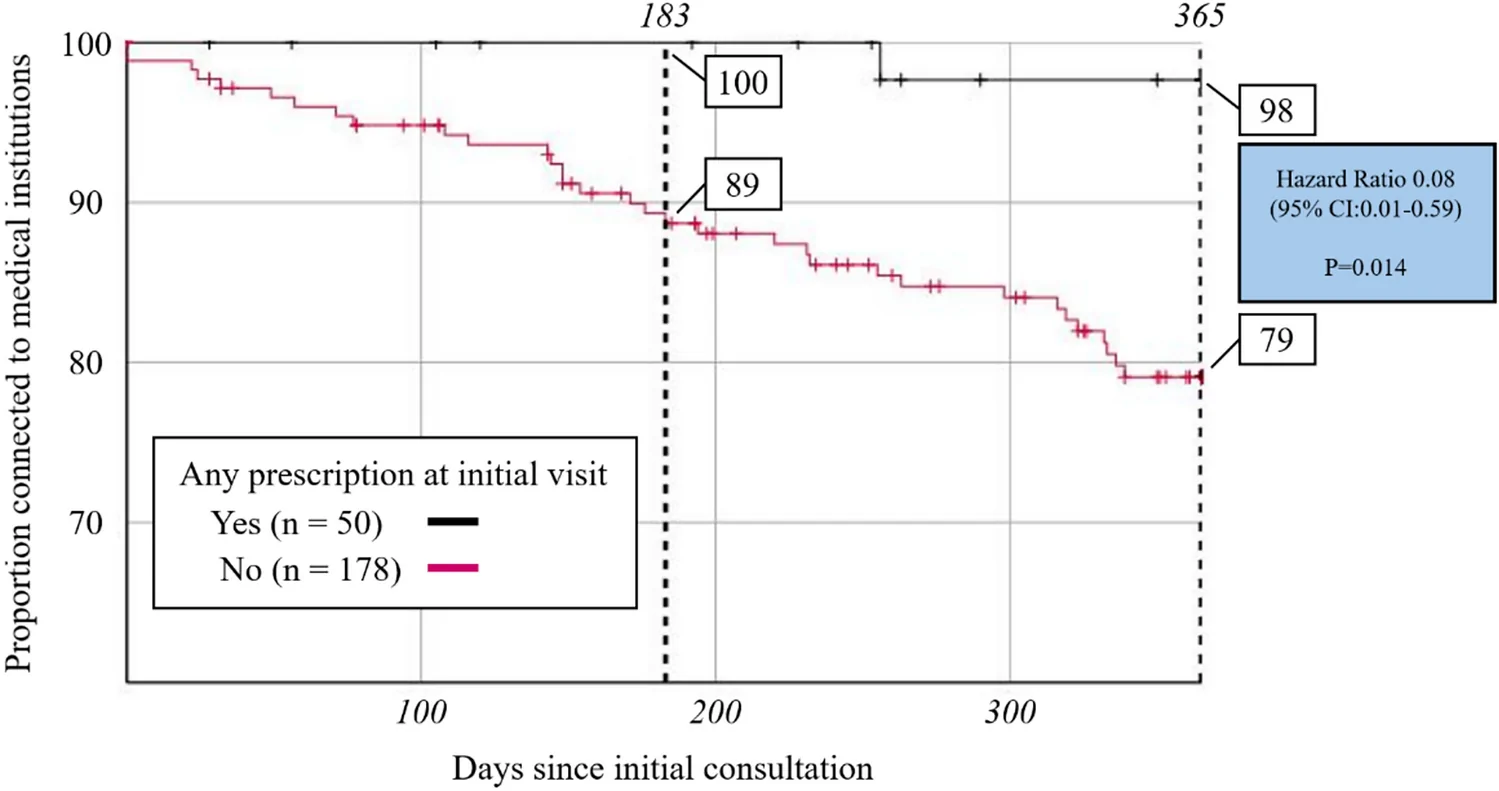
Kaplan–Meier survival curve for the period from treatment to dropout (female). CI, confidence interval

The Kaplan–Meier survival curve for female patients from initial treatment to dropout is shown in Fig. 3. A significant difference was observed between patients who received any prescription at the initial visit and those who did not (HR = 0.08, 95% CI: 0.01–0.59, p = 0.014).

## Discussion

The present study reveals key findings regarding treatment dropouts in child and adolescent psychiatry. Multivariate logistic regression analysis showed that children from divorced or separated families were more likely to discontinue treatment, whereas those who were prescribed medications at the initial visit were less likely to drop out. Cox proportional hazards analysis further supported the protective effect of the initial medication prescription against treatment dropout over time. The Kaplan–Meier survival analysis demonstrated that patients who were initially prescribed medication had significantly higher treatment adherence rates at 1 year, with a particularly pronounced effect in female patients. These findings align with those of previous research indicating that both family structure and the initial treatment approach significantly influence treatment adherence in pediatric psychiatric populations. For instance, a study that maximized pharmacological treatment adherence in children and adolescents found that telehealth applications effectively improve medication adherence (Karabulut et al., 2023). However, our study uniquely highlights the interplay between family dynamics, initial prescription practices, and long-term treatment adherence, providing a more comprehensive understanding of factors influencing dropout rates in child and adolescent psychiatry (Edgcomb & Zima, 2018; Raman et al., 2015).

Multivariate logistic regression analysis revealed significant associations between specific factors and treatment dropouts in child and adolescent psychiatry. Notably, children from divorced or separated families were 2.28 times more likely to discontinue treatment. Conversely, patients who received medication at their initial visit were less likely to drop out, with an OR of 0.27. These findings align with those of previous studies and offer new insights. Our study extends this knowledge by quantifying the substantial impact of parental divorce or separation on the treatment dropout risk. The protective effect of the initial medication prescription against dropouts was particularly noteworthy. This finding aligns with that of Gajria et al. (2014), who reported that medication type influenced adherence; however, our study uniquely highlights the strong protective effect of any initial medication prescription (Dinnissen et al., 2020). This suggests that early pharmacological interventions may play a crucial role in maintaining treatment engagement, potentially due to rapid symptom alleviation or increased perceived treatment efficacy. Our results contribute to the existing literature by emphasizing the interplay between family dynamics and initial treatment approaches in predicting dropout rates (Kalaman et al., 2023), offering valuable insights into clinical practice in child and adolescent psychiatry.

In Japan, the cultural norm of patients visiting medical institutions to obtain prescriptions may explain outcomes, as many medications that are sold over the counter in other countries require prescriptions in Japan, and the universal health insurance system significantly reduces the financial burden of medical expenses for patients. In this context, psychiatric services for children and adolescents are mainly provided through hospital-based outpatient clinics and are generally regarded as a legitimate route to obtaining professional support and medication, although stigma toward mental illness still persists. Referrals from schools and pediatricians are relatively common, and this healthcare culture may partly explain why receiving an initial prescription was associated with lower dropout rates in our study.

Additionally, time-to-event analysis using the Cox proportional-hazards model revealed significant associations between the initial prescription and treatment dropout over time. Notably, patients who received any prescription at their initial visit were 0.28 times less likely to drop out of treatment during the follow-up period. This HR indicated that the initial medication prescription substantially increased the likelihood of continued treatment engagement.

These findings align with those of previous studies and offer new insights. Gajria et al. (2014) found that medication type influenced adherence in patients with attention-deficit/hyperactivity disorder; our study extends this finding by demonstrating the strong protective effect of any initial medication prescription against dropout. Similarly, Blader (2004) reported that early treatment decisions significantly affect long-term adherence in pediatric psychiatric populations. Our results are consistent with those of a recent study by Örengül and Görmez (2017) that emphasized the importance of early intervention in maintaining treatment engagement. However, our study uniquely quantifies the magnitude of this effect in children and adolescents. Furthermore, although Cheung et al. (2021) discussed the complexities of psychotropic prescriptions in children, our findings suggest that the initial medication may serve as a crucial engagement tool, potentially increasing treatment adherence, regardless of the specific medication prescribed. This study provides a time-dependent analysis of the relationship between initial prescription practices and long-term treatment adherence in children and adolescents.

Nevertheless, the indiscriminate use of drugs should be avoided. In child and adolescent psychiatric care, psychosocial interventions should be the primary treatment choice rather than pharmacotherapy (Comer et al., 2013; Shrestha et al., 2020). Medication therapy alone tends to contribute to dropouts, highlighting the importance of combining medication with psychosocial interventions to sustain treatment engagement (Örengül & Görmez, 2017).

Moreover, Kaplan–Meier survival analysis revealed a significant impact of the initial prescription on treatment adherence in child and adolescent psychiatry. Overall, patients who received any prescription at their initial visit were 0.28 times less likely to drop out of treatment. This effect was more pronounced in female patients, with an HR of 0.08. After 1 year, 93% of patients who received an initial prescription remained in treatment, compared to 79% of those who did not. This difference was particularly striking among female patients, with 98% retention for those initially prescribed medication versus 79% for those who were not. These HRs indicated that initial medication prescriptions substantially increased the likelihood of continued treatment engagement, especially in female patients. Taken together, these findings mean that, in everyday clinical practice, prescribing medication at the first visit—when clinically appropriate—may considerably reduce the risk of early treatment dropout, particularly among girls, even for readers who are less familiar with survival analysis statistics. Initial non-adherence to antidepressants, especially selective serotonin-reuptake inhibitors, increases the number of sick days taken by depressed patients, leading to a deterioration in their health and an increase in social costs (Aznar-Lou et al., 2018). Furthermore, research emphasizes the importance of starting drug therapy early for patients with first-episode psychosis, and early intervention has been shown to contribute to successful treatment (Abdel-Baki et al., 2012).

Although this study was conducted within the Japanese healthcare system, several implications may be applicable to other settings and cultures. When clinically appropriate, carefully considered early pharmacological interventions combined with clear psychoeducation may help to enhance treatment engagement and reduce early dropout. In addition, the increased dropout risk among children from divorced or separated families highlights the importance of proactively identifying and supporting high-risk family constellations to promote continuity of care.

### Study Limitations

The generalizability of the findings of this study remains unknown. Given that the research was conducted in a single district, the result may not be representative of the entire population of children and adolescent psychiatric patients. Additionally, the retrospective nature of the study may have introduced potential bias in data collection and interpretation. Moreover, there was a marked imbalance in sample size between the dropout and non-dropout groups, which may have reduced the statistical power to detect smaller effects and limited the precision of some estimates. In addition, we did not use objective measures of medication adherence. Although treating psychiatrists routinely asked patients and caregivers about medication use at follow-up visits, we did not perform pill counts or inspect medication bottles, so our findings regarding prescriptions may not fully capture actual medication intake. We also did not systematically assess whether attendance at follow-up visits was primarily driven by patients themselves or by their family members. Therapist-related factors were not considered, despite their known influence on dropout rates. In particular, physicians with greater experience or stronger therapeutic skills may be more likely to prescribe at the first appointment while also fostering better treatment engagement, which could mean that the protective association of an initial prescription partly reflects physician characteristics rather than the prescription itself. Furthermore, the study focused on outpatient settings, thereby reducing its applicability to inpatient or community-based psychiatric care. The 1-year follow-up period may not capture long-term treatment outcomes or patterns of dropout beyond this timeframe.

## Conclusions

This study provides valuable insights into the factors that influence treatment dropouts in children and adolescents with psychiatric disorders. The findings highlight the significant impact of initial medication prescriptions on treatment adherence, particularly among female patients. Parental divorce or separation has been identified as a risk factor for treatment dropout, emphasizing the importance of family dynamics in treatment planning. These results suggest that early pharmacological interventions and targeted support for patients from divorced or separated families may improve treatment retention rates. Future research should aim to develop and evaluate interventions to address these specific risk factors and to enhance long-term treatment adherence in child and adolescent psychiatric care.

## Data Availability

No datasets were generated or analysed during the current study.

## Declarations

### Use of Generative AI

The authors used a generative artificial intelligence tool to assist with language editing and phrasing of the manuscript. All content was critically reviewed, verified, and approved by the authors, who are fully responsible for the final version of the manuscript.
